# Optimizing microwave ablation planning with the ablation success ratio

**DOI:** 10.1038/s41598-025-94957-4

**Published:** 2025-03-26

**Authors:** Christina A. Neizert, Hoang N. C. Do, Miriam Zibell, David Sinden, Christian Rieder, Jakob Albrecht, Stefan M. Niehues, Kai S. Lehmann, Franz G. M. Poch

**Affiliations:** 1 Department of General and Visceral Surgery, Charité-Universitätsmedizin Berlin, Corporate Member of Freie Universität Berlin and Humboldt-Universität zu Berlin, Hindenburgdamm 30, 12203 Berlin, Germany; 2 Landesamt für Gesundheit und Soziales, Turmstraße 21, 10559 Berlin, Germany; 3 Fraunhofer Institute for Digital Medicine MEVIS, Max-Von-Laue-Straße 2, 28359 Bremen, Germany; 4 Department of Radiology, Caritas-Klinik Dominikus, Kurhausstraße 30, 13467 Berlin, Germany

**Keywords:** Surgical oncology, Cancer therapy

## Abstract

The size of hepatic microwave ablations (MWA) is often difficult to predict due to cooling effects from liver vessels. This study introduces a simplified predictive model, the Ablation Success Ratio (ASR), which estimates the likelihood of a successful ablation based on tumor size and specific ablation parameters. The ASR model is based on the three-dimensional minimum ablation radius (r_3Dmin_), defining the spherical region within which complete ablation is achieved. To validate the ASR, standardized MWAs were performed in an ex vivo porcine liver model using a glass tube to simulate the vascular cooling effect. Ablations (*n* = 148) were conducted at 100 W for 5 min, with antenna-to-vessel (A-V) distances set at 2.5, 5.0, and 10.0 mm. Subsequently, the r_3Dmin_ was calculated. Without vascular cooling (0 ml/min, corresponding to an intraoperative Pringle maneuver), an ASR of 100% was achieved for ablation diameters up to 20 mm. However, in the presence of vascular cooling (1–500 ml/min), the ASR reached 100% only for ablation diameters up to 12 mm, demonstrating that the ASR effectively includes the impact of vascular cooling effects. The ASR is a promising and simple approach for predicting ablation success while also accounting for vascular cooling effects in hepatic MWA.

## Introduction

Primary and secondary liver malignancies like hepatocellular carcinoma (HCC) or colorectal liver metastases (CRM) are among the most common tumor diseases worldwide^1,2^. In addition to surgical resection, minimally invasive thermal ablation procedures are potentially curative treatment options if tumor size and location are suitable^3–5^. In clinical practice, hepatic microwave ablation (MWA) has been established among other thermoablative procedures such as radiofrequency ablation (RFA)^6^. With MWA, higher temperatures in the ablation center and therefore larger and more uniform ablations are achieved compared to other in situ procedures^3^. MWA is also less susceptible to cooling effects of naturally occurring liver vessels than RFA, as propagation into the tissue depositions energy into the tissue, rather than be driven by heat diffusion^3,7,8^. However, studies have shown that vascular cooling occurs in MWA as well, suggesting that further research is required^9–11^.

**Fig. 1:**
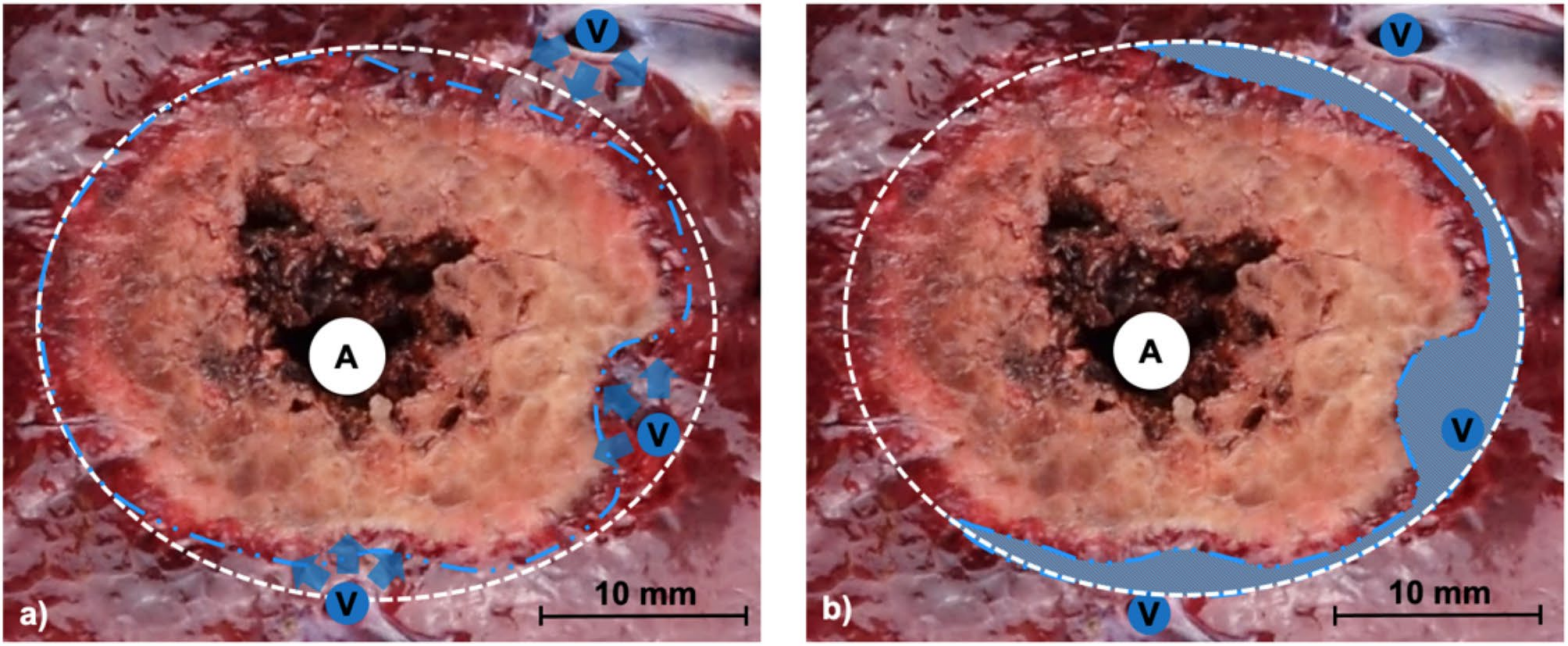
(**a**) Exemplary macroscopic cross-section of an ablation, showing the MW-antenna (A) and adjacent liver vessels (V). The predicted ablation zone without any cooling effects was outlined with a white dotted line. (**b**) Liver vessels (V) withdraw heat from the ablation area, leading to the so-called “heat sink effect” (shaded area). This results in flattening or narrowing of the ablation zone (blue dotted line), which likely affects ablation success.

The disadvantage of thermal ablations such as MWA is that there is no postinterventional histopathologic confirmation equivalent to the “R0 situation” after surgical tumor resection^12^. Technical success can only be evaluated indirectly by imaging techniques such as contrast-enhanced computed tomography (CECT), contrast-enhanced ultrasound (CEUS) or magnetic resonance imaging (MRI)^13,14^. Variability in imaging protocols across institutions can lead to inconsistencies in assessing the actual ablation volume, potentially resulting in over- or underestimation. Additionally, the absence of direct visibility in postinterventional imaging may make it difficult to detect small tumor residues^13,15^. Moreover, cooling effects make a prediction of ablation success particularly difficult^9–11^ (Fig. 1).

Therefore, MWA need to be planned beforehand as accurately as possible in clinical routine. Software-based numerical simulations are being utilized to estimate ablation size^16–18^. However, the number of variables influencing MWA such as manufacturers data, liver tissue properties (tumor, cirrhosis, hepatic steatosis, etc.), liver vessels including possible cooling effects, as well as tumor localization and size present a major challenge in accurately predicting ablation size^16,18–20^. Although several navigation software systems are available outside of research projects that enable patient-specific calculation of MWA, there is an unmet clinical need for a simplified, robust and easily applicable predictability algorithm.

We aimed to develop a score (Ablation Success Ratio - ASR) which specifies the probability of ablation success in relation to tumor size based on real ablation data. Usually, only absolute values for the expected ablation size depending on the selected ablation parameters are provided by the manufacturer^21,22^. However, it has been shown that ablation sizes are subject to fluctuation due to the cooling effect of liver vessels^11,19,23^. Eventually, the overall goal for the ASR is to take into account natural variations in ablation size by incorporating MWA of patients retrospectively. Beforehand, an ex vivo validation of the ASR is necessary.

The aim of this study was to introduce and evaluate a new score (ASR) for the prediction of hepatic microwave ablation considering vascular cooling effects using a standardized ex vivo experimental setup.

## Results

A total of 148 microwave ablations were performed in ex vivo porcine livers. Twenty-two ablations were repeated due to naturally occurring large liver vessels (*n* = 10), technical errors resulting in automatic ablation termination (*n* = 3) and the ablation extending beyond the liver sample (*n* = 9). Consequently, 126 ablations yielding 1498 individual slices were evaluated. A qualitative, quantitative (ablation volume) and semi-quantitative analysis of these ablations has already been published^11^: We could show that although a cooling effect around the vessel occurred macroscopically in almost all ablations with perfusion, a decrease in ablation volume was detected only at the maximum flow rate of 500 ml/min at an antenna-to-vessel distance (A-V distance) of 2.5 mm (*p* = 0.002). In all other test series, no difference in ablation volume was observed between ablations with and without perfusion of the glass tube (*p* > 0.05)^11^. Therefore, a sole assessment of the ablation volume seems insufficient to determine the extent of vascular cooling effects. We therefore further analyzed three-dimensional ablation radii (r_3D_) in this study as an additional parameter to examine cooling effects in MWA.

In contrast to the ablation volume, vascular perfusion had an impact on the minimal ablation radius in all three test series (Table 1). In particular, the position of the vessel within the ablation had an influence on the 3D minimal ablation radius (r_3Dmin_). A radius reduction already occurred at the lowest flow rates (≥ 1 ml/min) when the vessel was localized at the ablation edge. In contrast to the minimum ablation radius, vascular perfusion or vessel position in relation to the ablation center had no influence on the maximum ablation radius.

**Table 1:** 3D ablation radii (minimum and maximum in mm) for each A-V distance and all flow rates (median (min / max); *p ≤ 0.008; ^(^*^)^*p* ≥ 0.008 - ≤ 0.05.

	0 ml/min	1 ml/min	2 ml/min	5 ml/min	10 ml/min	100 ml/min	500 ml/min
A-V distance: 2.5 mm							
r_min_	9.3 (8.7/10.4)	9.3 (7.5/9.9)	8.2^(^*^)^ (5.6/9.5)	7.6^(^*^)^ (7.1/8.9)	6.7* (4.8/8.3)	5.8 * (4.3/6.9)	5.3 * (4.8/6.0)
r_max_	15.4 (13.5/16.9)	16.0 (14.5/18.1)	14.3 (12.8/14.5)	14.2 (13.4/16.2)	14.9 (14.2/20.2)	14.5 (13.5/15.3)	14.4 (13.5/16.2)
A-V distance: 5.0 mm							
r_min_	9.2 (8.4/10.7)	8.3 (6.6/9.8)	5.9^(^*^)^(5.0/9.6)	5.4*(3.5/5.7)	4.9*(4.6/5.9)	4.2* (3.9/4.9)	4.8* (3.7/6.9)
r_max_	14.7 (12.8/18.4)	15.3 (12.9–16.1)	16.4 (14.9/20.2)	14.3 (12.4/15.6)	13.4 (11.4/16.9)	14.5 (12.1/16.8)	14.6 (12.6/17.5)
A-V distance: 10.0 mm							
r_min_	8.8 (8.7/10.5)	8.5 (6.9/9.6)	7.0* (6.5/7.5)	7.0* (6.6/8.4)	7.0* (5.7/7.9)	6.6* (6.2/7.5)	6.4* (5.5/7.2)
r_max_	15.8 (12.8/18.3)	15.6 (13.7/16.8)	16.2 (14.8/16.8)	16.4 (13.1/18.3)	16.3 (12.0/20.1)	16.9 (14.4/18.8)	14.6 (12.2/18.1)

**Fig. 2:**
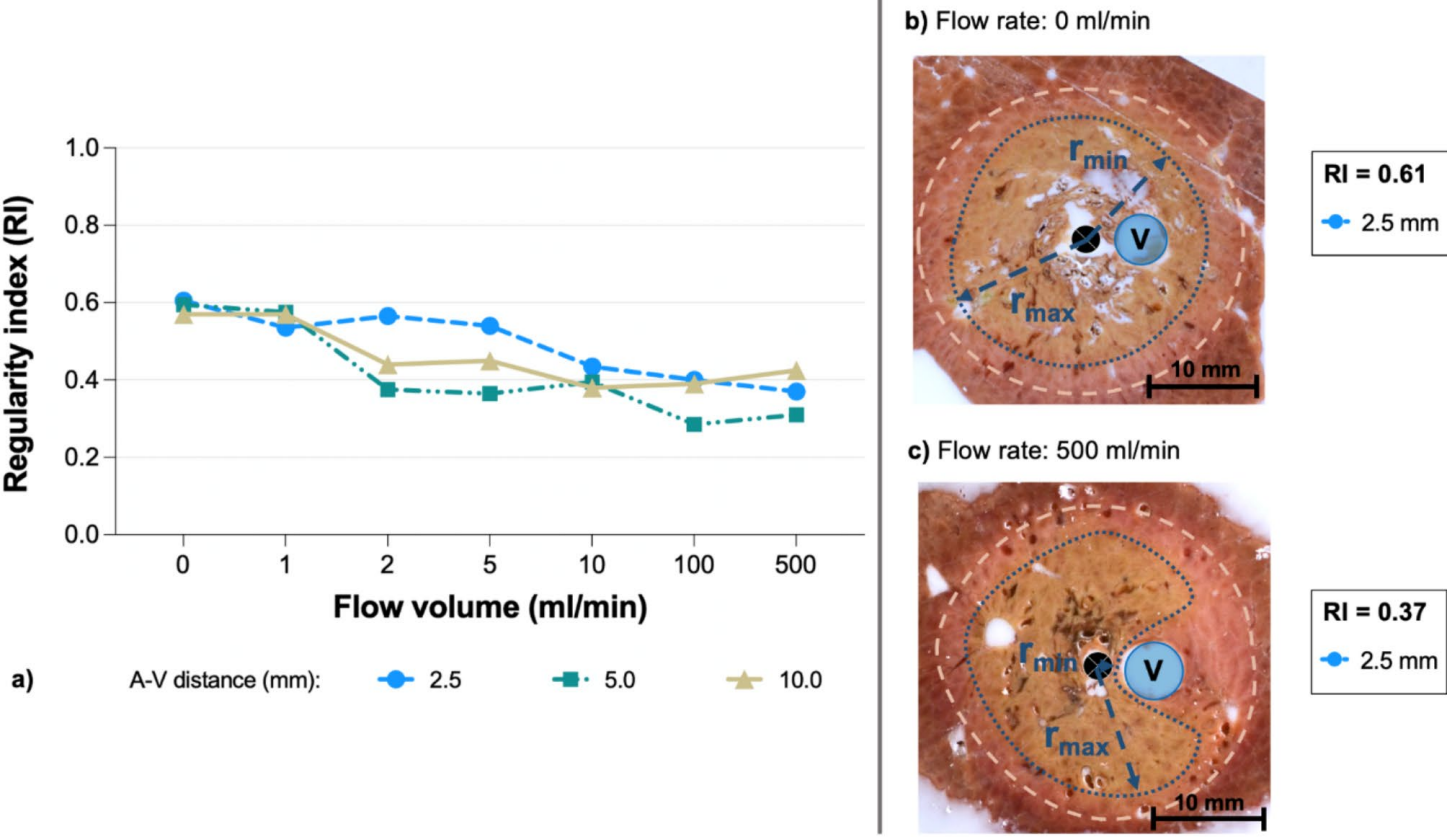
(**a**) 3D RI for all ablations in dependence of the vascular flow rate and A-V distance. (**b**) At an A-V distance of 2.5 mm the RI was 0.6 when there was no flow (0 ml/min). The maximal cross-sectional diameter was used exemplarily. Outlines of the WZ (blue dotted) and RZ (white dashed) as well as the vessel (V) and ablation center (black dot) are shown. (**c**) At a maximal flow rate of 500 ml/min ablation geometry was irregular. Ablations were indented and showed a pronounced cooling effect at a close A-V distance of 2.5 mm (RI 0.4).

The three-dimensional regularity index (RI) was used to describe ablation geometry (Fig. 2). The RI for ablations without vessel perfusion (0 ml/min) was around 0.6. This indicated that ablations already had an ellipsoid shape even without any influence of vascular cooling. The RI decreased further with increasing flow rates and was about 0.4 at a maximum flow rate of 500 ml/min. MWA are therefore already non-circular without any cooling effects. However, the vascular cooling effect has an additional impact on ablations and consequently must be taken into account when planning MWA.

### Ablation success ratio

**Fig. 3:**
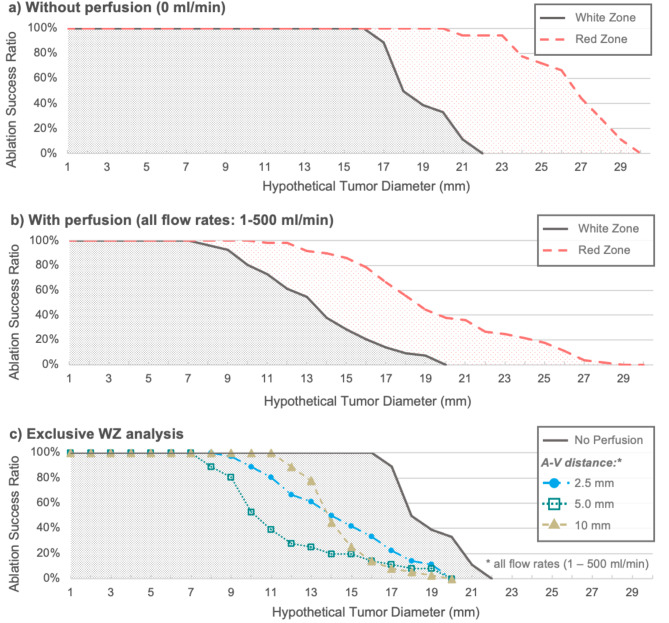
Ablation success ratio (ASR) in dependence to hypothetical tumor size (mm) for ablations without (**a**) or with (**b**) vascular perfusion for both WZ and RZ based on ex vivo liver data. (**c**) Exclusive WZ analysis of the ASR for different A-V distances with vascular perfusion (1–500 ml/min). All ablations were carried out at 100 W for 5 min.

Figure 3 shows the ASR using the results of these ex vivo experiments. Ablation success is shown in relation to ablation size (mm). The x-axis represents a hypothetical tumor, which corresponds to the tumor to be ablated in clinical routine. Usually, an ablation consists of two zones: the inner White Zone (WZ; immediate cell death) and the Red Zone (RZ; partial cell death), which transitions into native liver tissue^13^. For this reason, both the WZ and RZ are shown in Fig. 3a/b. Figure 3a shows the ablation results without perfusion (0 ml/min) while Fig. 3b shows the ablations with perfusion (1-500 ml/min). As expected, the RZ is larger than the WZ in both plots. In the experiments without a cooling effect, the ASR was 100% up to a hypothetical tumor diameter of 20 mm in the RZ and 16 mm in the WZ. In contrast, the tumor diameter at which a safe ablation (ASR = 100%) can be assumed, decreased considerably for test series with perfusion (RZ: 12 mm, WZ: 7 mm). The ASR also varied depending on the A-V distance (Fig. 3c). Ablation success was noticeably lower when the vessel was located near the ablation margin (5 mm). As the distance between the vessel to the ablation border increased (10 mm), the ASR improved accordingly. In summary, our experimental ex vivo trial in native porcine liver (100 W, 5 min) showed that safe ablation was possible for tumors with a diameter of 12 mm (RZ) or 7 mm (WZ) respectively, when there was vascular perfusion. In the absence of liver perfusion (corresponding to an intraoperative Pringle maneuver), safe ablation extended to tumor diameters of 20 mm (RZ) or 16 mm (WZ). MWA of larger tumors must be considered critically and should be assessed individually depending on the vicinity of the tumor to larger hepatic vessels. Safety distances around the tumor need to be regarded additionally in clinical practice when planning MWA.

## Discussion

Thermal ablations such as microwave ablation (MWA) are influenced by vascular cooling effects^3,7,24^. The extent of these cooling effects and therefore the exact ablation volume are difficult to predict. The aim of this study was the introduction and first evaluation of an innovative prediction score called “Ablation Success Ratio - ASR” for the planning of hepatic microwave ablation. We demonstrated that the ASR indicates ablation size taking into account vascular cooling effects in a standardized ex vivo setting. Depending on the applied ablation system and selected ablation parameters, clinicians may use the ASR in the future to decide whether complete tumor ablation is feasible.

After surgical resection of hepatic tumors, complete excision is confirmed by histological analysis. This is not possible in MWA due to the in-situ approach. Instead, technical success is determined indirectly using ultrasound, CT or MRI imaging. Precise pre-therapeutic treatment planning plays a particularly important role as imaging modalities are limited in their accuracy^21^. Ablation procedures are usually planned based on recommendations provided by the manufacturer. Depending on the ablation system and tumor diameter, specific ablation parameters are selected^21,22^. These manufacturer’s specifications normally refer to ablations that were performed under ideal conditions (ex vivo with the absence of vascular cooling effects). However, individual blood vessels in the liver, which transfer thermal energy away from the ablation site and therefore lead to vascular cooling effects, are not considered. For this reason, the manufacturer’s specifications tend to overestimate ablations in patients^21,22^. This may result in incomplete ablation and thus tumor recurrence. Therefore, vascular cooling effects of the patient-specific liver vasculature in relation to the antenna position must be considered when planning MWA. Software-based numerical simulations are able to calculate ablation size in advance including cooling effects, liver tissue properties as well as the ablation system and energy parameters^16–18,25,26^. However, these simulations require a high computational power and are too time-consuming for daily use in clinical practice^19,27^. Neither the manufacturer’s specification nor numerical simulations currently seem suitable for clinical routine to reliably anticipate ablation size. For this reason, a simplified method, which indicates ablation success is necessary.

The ASR is intended to close the gap between the simplified manufacturer’s recommendations and the complex numerical simulations. It is supposed to provide the clinician with a practical tool for predicting ablation success. Due to vascular cooling effects, ablations in vivo are rarely round, but often irregularly shaped. Although the minimum and maximum diameter of an ablation are often specified for a retrospective assessment, this information is insufficient for ablation planning due to the irregular occurrence of ablation shape^18,19,28,29^. For the clinical user, a fixed area around the antenna must be defined in which a safe ablation can be assumed. The antenna can then be placed in such a way that complete tumour ablation is ensured. Such an area is represented by the three-dimensional minimum radius (r_3Dmin_) of an ablation, which consequently is the basis of the ASR. The intended ablation size is set in relation to the number of MWA that have been performed, resulting in the probability of ablation success rather than an absolute value. By definition, the ASR ranges from 0 to 100%. For example, the ASR for very small tumors is close to 100%, whereby this value decreases with increasing tumor diameter. If required, a safety distance of 5–10 mm can be added.

For an initial validation, the ASR was developed using a standardized ex vivo model in this study. For clinical application of the ASR, a large number of ablations need to be evaluated. This could be implemented by assessing minimal ablation radii of patients retrospectively based on CT and MRI data, taking into account the respective ablation system, the applied energy input and the exposure duration. The quality of the ASR will hereby increase with the number of ablations analyzed. The ASR is close to clinical reality, as it takes ablation size variation depending on cooling effects and liver tissue properties into consideration. Additionally, a subgrouping for special cases such as perivascular tumors or tumors near the liver capsule as well as ablations with a Pringle maneuver can be included. However, it must be considered that the ASR is only applicable for the selected ablation setting (ablation system, ablation time, applied energy) and must therefore be determined again for all other cases with different ablation parameters.

Eventually, an individual ablation simulation that incorporates tumor location, vessel vicinity as well as tissue properties of the patient is the desired goal. Until this is implementable in clinical routine, preinterventional ablation planning should be based on ablation success derived from a retrospective analysis of real ablations rather than on manufacturer’s specifications.

In the present experimental ex vivo setting, safe ablations (ASR = 100%) were possible up to 20 mm (RZ) or respectively 16 mm (WZ) with the absence of liver perfusion and up to 12 mm (RZ) and 7 mm (WZ) with preserved liver perfusion. These results are not sufficient for clinical application, especially if a safety margin of 5 mm around the tumor is added. However, low energy parameters (ablation power: 100 W; ablation time: 5 min) and thus small ablation sizes were chosen in this experimental setup so that the ablations could be performed in the narrow porcine liver. This approach was reasonable as we did not want to investigate the absolute ablation size but the ASR in relation to vascular cooling effects. In clinical application, larger ablations can be expected because higher ablation parameters are used^30,31^. Therefore, our experimental ablation sizes should not be directly transferred to MWA in patients. Furthermore, a classification of an ablation into WZ and RZ is only applied macroscopically and histologically^13,32^. In clinical routine, this classification plays a subordinate role, as MWA is primarily evaluated using imaging techniques, which do not permit a color- or structure-based distinction between WZ and RZ^6^. Accuracy is further constrained by spatial and contrast resolution to approximately 2–3 mm, depending on the imaging modality used^33^. However, studies have shown that there is close conformity between the RZ and the ablation detected in CECT^15^. Since complete cell death is uncertain in the RZ, it is essential to include values for both WZ and RZ in experimental studies to accurately assess MWA^28^. Our experiments showed a Regularity Index (RI) of approximately 0.6 for ablations performed without vessel perfusion (0 ml/min), indicating an ellipsoidal rather than perfectly round ablation shape, even in the absence of vascular cooling. In clinical studies, the RI is derived from CT or MRI measurements, where WZ and RZ cannot be clearly distinguished^12^. This often results in RI values closer to 1.0, as the RZ is included in the measurement^12,34^. Unlike other studies, we focused exclusively on the WZ, which generally conforms to the shape of the ablation probe, leading to a more elongated appearance. Consequently, our RI values are lower compared to other research groups and cannot be directly translated to clinical practice. Intrinsic factors inherent to the ablation process such as uneven energy distribution of the ablation device or different thermal properties of the liver tissue (cirrhosis, tumor, neoadjuvant therapy, etc.) may contribute to lower RI values^12,35^. Additionally, it must be noted that a large number of ablations is required for the ASR to increase in quality and to reduce the uncertainty of values due to naturally occurring variations in tissue properties. The implementation of a database with the help of software programs seems advisable in this case. Further limitations of the experimental setup include the use of a glass tube instead of natural liver vessels, the absence of a liver tumor and a macroscopic ablation analysis instead of using imaging techniques. The use of a glass tube as a vessel is very well established in experimental studies^9,11,36^. It is known that glass has similar heat properties than blood vessels and therefore is a suitable substitute in an ex vivo models^37^. In our study only one vessel was utilized to induce cooling effects, so no conclusion can be drawn regarding the effects of very large vessels or the complex vascular condition present in clinical practice in general. We observed a greater decrease in ablation success when the vessel was situated at the ablation margin. As the outer ablation margin is characterized by the lowest energy density, it is particularly vulnerable to the cooling effect. Since the ASR automatically considers vascular cooling effects, an application of the ASR with in vivo and/or clinical ablations seems possible. Although tumor models for HCC exist, we used native ex vivo porcine liver for an initial validation of the ASR due to ethical and cost-effective reasons^38^. Moreover, blood-perfused tissue models are commonly utilized for evaluating MWA^26,39,40^. Physical characteristics of human and porcine liver as well as tumor tissue are known, so that a translation to a tumor model is feasible with the aid of numerical simulations^41^. This study focused on healthy liver tissue that was analyzed immediately after MWA, limiting the ability to assess long-term changes in ablation zones. Studies indicate that the inner red zone progressively becomes non-viable^42^. Long-term studies are essential to better understand how ablation zones change over time. Our experiments were conducted at room temperature. Due to the higher thermal gradient compared to body temperature, cooling effects may be more pronounced. Previous studies on radiofrequency ablation (RFA) have shown that macroscopic results at room and body temperature are generally comparable^36^. Lastly, we solely assessed MWA macroscopically in our study according to Mulier et al.^43^. In clinical routine the ASR will be based on real ablations and therefore depend on an evaluation using imaging modalities. It has to be considered that digital ablation assessment is affected by artifacts, hemorrhage, cooling effects and tissue edema^44^. Tissue shrinkage from dehydration and protein denaturation above 60 °C may further underestimate an ablation evaluation^45–47^. In our study, a macroscopic approach was deliberately chosen to develop and test the ASR under standardized conditions. The implementation of the ASR to imaging techniques in an in vivo setting is the next preferable step.

## Conclusion

The ASR is a promising tool for assessing ablation success in MWA preinterventionally taking into account tumor size, cooling effects of natural liver vessels and ablation parameters. For the use in clinical practice, the ASR should be based on a retrospective evaluation of real patient ablations.

## Materials and methods

### Definition of the ablation success ratio (ASR)

**Fig. 4:**
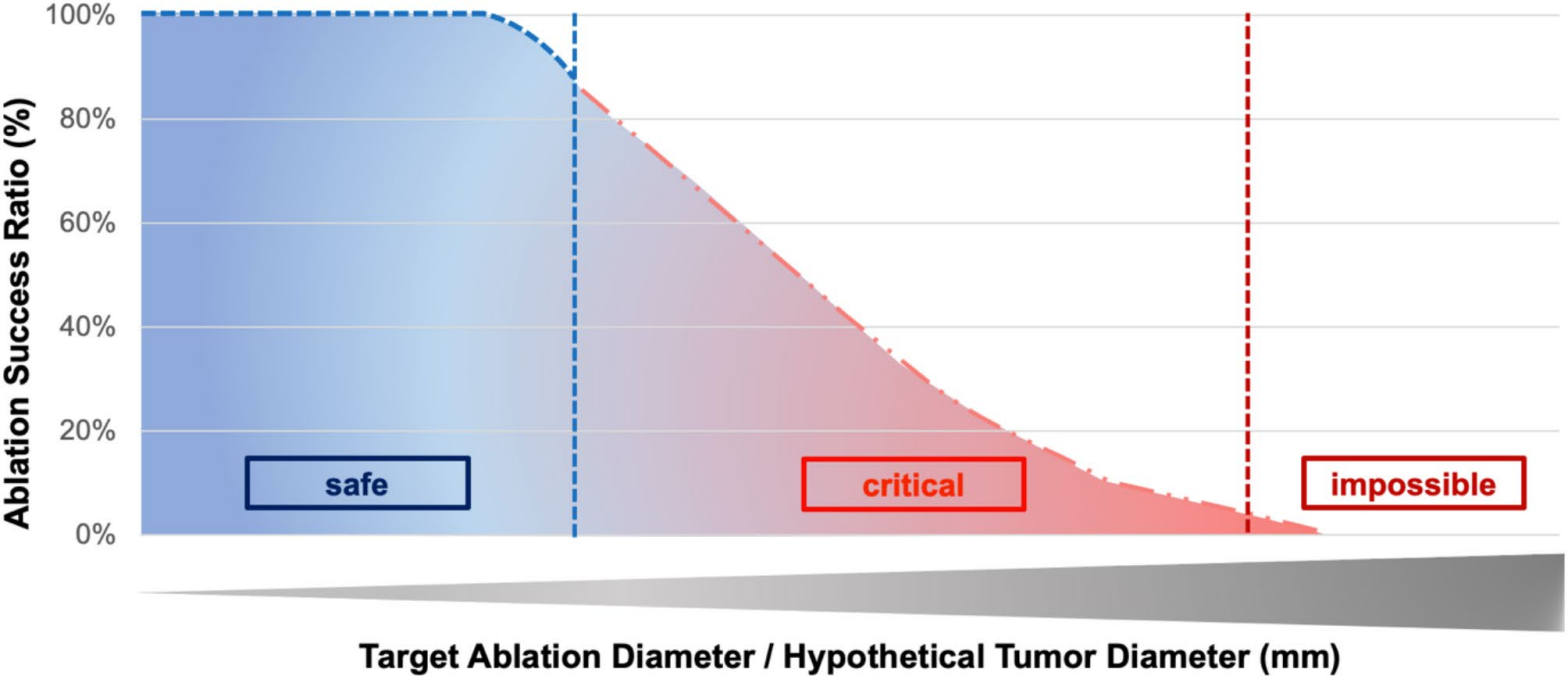
A conceptual representation of the ablation success rate (ASR) as it might be available to clinicians at the bedside in the future. This example graph shows how clinicians might use the ASR for tumor ablation, classifying success as “certain”, “critical” or “impossible”. The final visualization will be based on actual patient data.

The primary aim of this study is the establishment and validation of the methodology of the ASR in a standardized ex vivo experimental setup. The Ablation Success Ratio (ASR) is designed to predict the probability of achieving complete ablation depending on the given tumor diameter. Thermal ablations often result in irregularly shaped areas of tissue destruction. The ASR focuses solely on the minimum three-dimensional ablation radius (r_3Dmin_), which defines the spherical zone of tissue that has been completely ablated. This radius is crucial for planning an effective MWA. After an ex vivo validation (presented in this study), the goal is that the ASR will be derived from a retrospective analysis of real patient data (r_3Dmin_) in the future using standardized ablation zone measurements^15,28^. It will specifically examine cases where identical ablation parameters (MWA system, energy settings) were used. This data-driven approach ensures that the ASR reflects actual clinical outcomes. The ASR represents the percentage of these analyzed MWA cases in which the ablation area exceeded the planned target area required for complete tumor ablation. An ASR of 100% indicates that all analyzed ablations were larger than the target, signifying a “safe” ablation (Fig. 4). Conversely, an ASR of 50% suggests that only half of the ablations were larger than the target, warranting a more critical assessment of the planned procedure.

The ASR will determine the success of an ablation with a planned ablation diameter of ($\:x$) intended to treat a tumor with a specific diameter and is calculated as follows:

Given $\:{n}_{total}$ (total number of ablations analyzed) and $\:{r}_{3Dmin}$, the diameter of each minimal ablation ($\:{d}_{min})$ is:

$$\:{d}_{min}\:=\:{2\times\:r}_{3Dmin}$$

The percentage of ablations $\:{n}_{+}$ with diameters greater than $\:x$ is derived by:

$$\:ASR=\:\frac{{n}_{+}}{{n}_{total}}\:\times\:\:100$$

where $\:{n}_{+}$ is the number of ablations for which the diameter $\:{d}_{min}\:>x$.

The number $\:{n}_{+}$ can be formally expressed as the sum over all $\:{n}_{total}$ ablations:

$$\:{n}_{+}=\:\sum\:_{i=1}^{{n}_{total}}Heaviside(2\:\times\:\:{r}_{3Dmin,\:i}-x)\:$$

The Heaviside function $\:Heaviside\left(z\right)$ is a step function defined as:

$$\:Heaviside\left(z\right)=\:\left\{\begin{array}{c}1\:\:\:if\:z\:>0\\\:0\:\:\:if\:z\:\le\:0\end{array}\right.$$

The accuracy of the ASR will increase with the number of ablations analyzed ($\:{n}_{total}$). This method allows the calculation of the percentage of ablations exceeding the target size (ASR), facilitating a quantitative analysis of the effectiveness and efficiency of MWA relative to the desired target size.

### Validation of the ASR in an ex vivo study

**Fig. 5:**
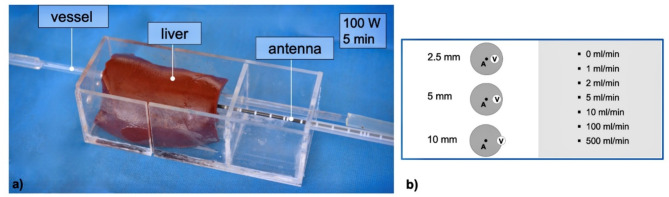
(**a**) Experimental setup using a custom-made aiming device which allowed exact placement of the antenna and vessel into the liver segment. (**b**) Three different antenna-to-vessel distances (A-V distance) were analyzed at seven different flow rates (ml/min) so that a total of twenty-one different set-ups were planned.

The aim of the experimental setup was to evaluate the ASR in relation to different positions of hepatic vessels with respect to the ablation zone under standardized conditions. All experiments were conducted in an established ex vivo model with native porcine livers obtained from an abattoir (Brandenburg, Germany) within six hours after slaughtering^9,11,48^. To induce standardized vascular cooling effects, a perfused glass tube was used as “liver vessel”. Seven different vascular flow rates were evaluated. MWA were performed in a custom-made aiming device that enabled the insertion of the vessel (glass tube) and exact positioning of the microwave antenna at three different distances into the liver (Fig. 5). After MWA, the ablations were cut into half and directly snap frozen. A 3D ablation evaluation was then carried out to validate the ASR. Details of the exact experimental setup are described below.

### Microwave ablation

The Emprint™ MWA system (Covidien, Boulder, CO, USA) with a 2.45 GHz generator was applied for all experiments. An antenna with a shaft length of 20 cm and an active tip length of 25 mm (Emprint™, Covidien, Boulder, CO, USA) was used. Internal cooling of the antenna was secured with saline solution and a continuous flow rate of 60 ml/min. A glass tube with an inner diameter of 3 mm and an outer diameter of 5 mm simulated a natural liver vessel^11,36,48^. This vessel was connected to a peristaltic pump (flow rates ≤ 5 ml/min: Minipuls^®^ 3, Abimed, GILSON, USA; flow rates ≥ 10 ml/min: Watson-Marlow™ 323E/D, Bredel Pumps, Falmouth, Cornwall, England). A custom-made aiming device made from acrylic glass ensured parallel placement of the microwave antenna (A) and vessel (V). Three different A-V distances were analyzed: 2.5, 5.0, 10.0 mm. Seven different flow rates were tested for each of the three A-V distances for 5 min at 100 W: 0, 1, 2, 5, 10, 100, 500 ml/min (Fig. 5), resulting in twenty-one different ablation settings (*n* = 6 ablations for each setting). Ablations were performed at room temperature. After MWA, ablations were halved along the maximum cross-sectional diameter, which was expected at the center of the active zone of the antenna. Following a tissue preparation with Tissue Tek^®^ O.C.T.™ (Sakura Finetek Germany GmbH, Staufen, Germany), ablations were snap frozen with liquid nitrogen and stored at -80 °C. A croystat (CryoStar™ NX70 Cryostat, ThermoFisher Scientific, Waltham, USA) was used to cut slices with a defined layer thickness of 50 μm from the respective ablation halves. Every 2 mm, the exposed plane was photographed next to a millimeter scale so that the corresponding plane could be included in a consecutive evaluation.

### Ablation analysis

**Fig. 6:**
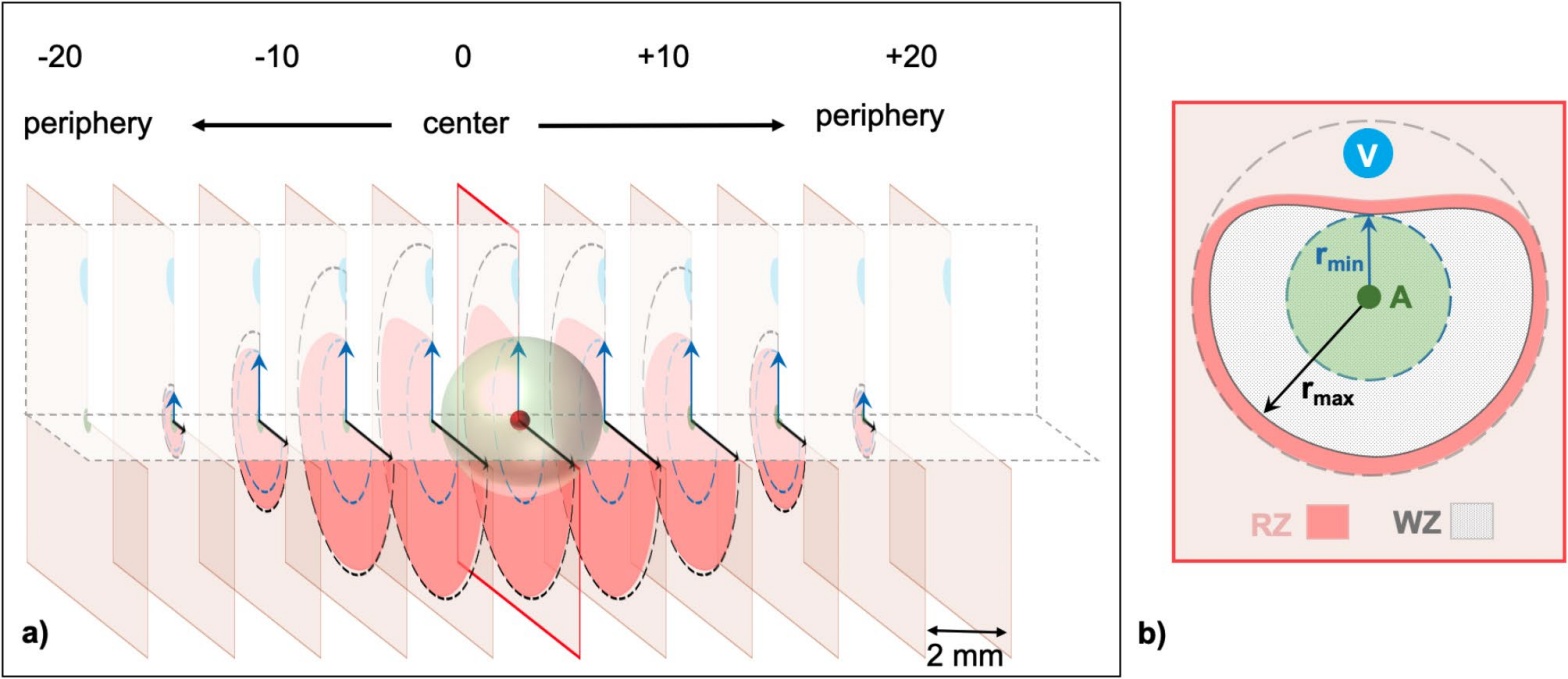
(**a**) Exemplary display of a segmented ablation. Initially, all ablations were bisected at level of the center of the active zone of the antenna to achieve the largest cross-sectional area. Starting from the center (0), each ablation half contained a maximum of ten additional segments (+/- 20). (**b**) The antenna (A) and vessel (V) were identified and the WZ and RZ outlined manually in each ablation segment. The software then calculated respective ablation radii. In clinical practice, a microwave ablation ideally covers the liver tumor completely (green sphere). Cooling effects, however, can lead to discrepancies of the expected minimal radius and the actual minimal radius (cf. Fig. 1).

Images of all ablation slices were macroscopically analyzed with a custom-made software (MWANecrosisMeasurement, Fraunhofer Institute for Digital Medicine MEVIS, Bremen, Germany). First, the software calibrated the images using the photographed millimeter paper. Subsequently, the “white zone” (WZ) and “red zone” (RZ) were outlined manually based on the color differences of the ablated tissue compared to native liver parenchyma^32^. The WZ is defined by irreversibly damaged tissue and represents the area of the ablation where complete tumor destruction is expected^13,43^. It is macroscopically identified by its beige/grey color. Adjacent to the WZ is the reddish colored RZ, where tissue destruction is incomplete and tumor recurrence may occur^13^. Based on the manual outline, the software then computed the minimum (r_min_) and maximum (r_max_) radius of the WZ and RZ for each ablation layer/segment (2D) (Fig. 6).

### 3D ablation radii

Using the two-dimensional (2D) analysis from before, the minimum and maximum ablation radius of the entire ablation (three-dimensional, r_3D_) were subsequently calculated. The starting point for the calculation was the ablation center (C), defined by the antenna insertion point of the ablation plane with the largest ablation diameter. Next, the Pythagorean theorem was used to calculate the distance between the ablation center (C) and the minimum radius of the respective ablation plane (d_CR1_, d_CR2_, …, d_CR20_). This distance represented the hypotenuse in the Pythagorean theorem. The distance of the respective ablation plane from the ablation center (d_0mm_, d_2mm_, …, d_20mm_) and the corresponding minimum ablation radius (r_min1_, r_min2_, …, r_min20_) were used as the catheti of the right-angled triangle. Thus, the following formula was obtained: $\:{d}_{CRx}$ = $\:\sqrt{{{\text{r}}_{\text{m}\text{i}\text{n}\text{x}}}^{2}+\:{{\text{d}}_{\text{x}\text{m}\text{m}}}^{2}}$. The minimum radius of the entire ablation volume approximately represented the smallest value of the amount of all previously calculated minimum radii: r_3D_ = min (d_CR1_, d_CR2_, …, d_CR20_).

### Regularity index (RI)

Based on the previous results, the 3D minimum and maximum ablation radii were used to calculate a 3D regularity index (RI) of an ablation^13^. With the help of the RI, the ablation geometry was analyzed. The RI was established by the quotient of the 3D minimum and maximum radius (RI = r_3Dmin_ / r_3Dmax_). Values close to 1.0 correspond to an almost spherical ablation geometry, whereas values < 1.0 indicate ellipsoidal or irregular ablation shapes.

### Statistical analysis

Statistical analysis was performed using SPSS (IBM SPSS Statistics, version 29 for Windows, Armonk, USA). Data are expressed as median (minimum - maximum). The Kruskal-Wallis test was applied for analyzing multiple independent samples, while the Mann-Whitney U test was used for the comparison of two independent samples. A Bonferroni correction was included due to multiple testing. Therefore, the level of significance was set to *p* ≤ 0.008. p values ≥ 0.008 and ≤ 0.05 were not considered statistically significant but were interpreted as a trend.

## Data Availability

The datasets analysed in our study are available from the corresponding author on reasonable request.

## References

[1] Sung, H. et al. Global cancer statistics 2020: GLOBOCAN estimates of incidence and mortality worldwide for 36 cancers in 185 countries. *CA Cancer J. Clin.* **71**, 209–249. 10.3322/caac.21660 (2021). 33538338 10.3322/caac.21660

[2] Ferlay, J. et al. Estimating the global cancer incidence and mortality in 2018: GLOBOCAN sources and methods. *Int. J. Cancer*. **144**, 1941–1953. 10.1002/ijc.31937 (2019). 30350310 10.1002/ijc.31937

[3] Izzo, F. et al. Radiofrequency ablation and microwave ablation in liver tumors: an update. *Oncologist* **24**, e990–e1005. 10.1634/theoncologist.2018-0337 (2019). 31217342 10.1634/theoncologist.2018-0337 PMC6795153

[4] Vogel, A., Martinelli, E., clinicalguidelines@esmo.org, E. G. & Committee, E. G. C. E. a. Updated treatment recommendations for hepatocellular carcinoma (HCC) from the ESMO Clinical Practice Guidelines. *Ann Oncol* **32**, 801–805. 10.1016/j.annonc.2021.02.014 (2021). 10.1016/j.annonc.2021.02.014 33716105

[5] van der Lei, S. et al. Thermal ablation versus surgical resection of small-size colorectal liver metastases (COLLISION): an international, randomised, controlled, phase 3 non-inferiority trial. *Lancet Oncol.* **26**, 187–199. 10.1016/S1470-2045(24)00660-0 (2025). 39848272 10.1016/S1470-2045(24)00660-0

[6] Vogl, T. J., Nour-Eldin, N. A., Hammerstingl, R. M., Panahi, B. & Naguib, N. N. N. Microwave ablation (MWA): basics, technique and results in primary and metastatic liver Neoplasms - Review Article. *Rofo* **189**, 1055–1066. 10.1055/s-0043-117410 (2017). 28834968 10.1055/s-0043-117410

[7] Simon, C. J., Dupuy, D. E. & Mayo-Smith, W. W. Microwave ablation: principles and applications. *Radiographics* **25** (Suppl 1), 69–83. 10.1148/rg.25si055501 (2005). 16227498 10.1148/rg.25si055501

[8] Dodd, G. D., Dodd, N. A., Lanctot, A. C., Glueck, D. A. & rd, & Effect of variation of portal venous blood flow on radiofrequency and microwave ablations in a blood-perfused bovine liver model. *Radiology* **267**, 129–136. 10.1148/radiol.12120486 (2013). 23297326 10.1148/radiol.12120486

[9] Poch, F. G. M. et al. Cooling effects occur in hepatic microwave ablation at low vascular flow rates and in close proximity to liver Vessels - Ex vivo. *Surg. Innov.* **29**, 705–715. 10.1177/15533506221074619 (2022). 35227134 10.1177/15533506221074619

[10] Poch, F. G. et al. Periportal fields cause stronger cooling effects than veins in hepatic microwave ablation: an in vivo Porcine study. *Acta Radiol.* **62**, 322–328. 10.1177/0284185120928929 (2021). 32493033 10.1177/0284185120928929

[11] Neizert, C. A. et al. Three-dimensional assessment of vascular cooling effects on hepatic microwave ablation in a standardized ex vivo model. *Sci. Rep.* **12**, 17061. 10.1038/s41598-022-21437-4 (2022). 36224235 10.1038/s41598-022-21437-4 PMC9556636

[12] De Cobelli, F. et al. Microwave ablation of liver malignancies: comparison of effects and early outcomes of percutaneous and intraoperative approaches with different liver conditions: new advances in interventional oncology: state of the Art. *Med. Oncol.* **34**, 49. 10.1007/s12032-017-0903-8 (2017). 28220346 10.1007/s12032-017-0903-8

[13] Ahmed, M. et al. Image-guided percutaneous chemical and radiofrequency tumor ablation in an animal model. *J. Vasc Interv Radiol.* **14**, 1045–1052 (2003). 12902563 10.1097/01.rvi.0000083254.29749.e1

[14] Lubner, M. G. et al. Advanced CT techniques for hepatic microwave ablation zone monitoring and follow-up. *Abdom. Radiol. (NY)*. **47**, 2658–2668. 10.1007/s00261-021-03333-z (2022). 34731282 10.1007/s00261-021-03333-z PMC13155194

[15] Poch, F. G. et al. Immediate post-interventional contrast-enhanced computed tomography overestimates hepatic microwave ablation - an in vivo animal study. *Int. J. Hyperth.* **37**, 463–469. 10.1080/02656736.2020.1762936 (2020). 10.1080/02656736.2020.1762936 32396401

[16] Heshmat, A. et al. Using Patient-Specific 3D modeling and simulations to optimize microwave ablation therapy for liver cancer. *Cancers (Basel)*. **16**10.3390/cancers16112095 (2024). 10.3390/cancers16112095 PMC11171243 38893214

[17] Frackowiak, B. et al. First validation of a model-based hepatic percutaneous microwave ablation planning on a clinical dataset. *Sci. Rep.* **13**, 16862. 10.1038/s41598-023-42543-x (2023). 37803064 10.1038/s41598-023-42543-x PMC10558472

[18] Deshazer, G., Merck, D., Hagmann, M., Dupuy, D. E. & Prakash, P. Physical modeling of microwave ablation zone clinical margin variance. *Med. Phys.* **43**, 1764. 10.1118/1.4942980 (2016). 27036574 10.1118/1.4942980

[19] Young, S., Rivard, M., Kimyon, R. & Sanghvi, T. Accuracy of liver ablation zone prediction in a single 2450 mhz 100 Watt generator model microwave ablation system: an in human study. *Diagn. Interv Imaging*. **101**, 225–233. 10.1016/j.diii.2019.10.007 (2020). 31740266 10.1016/j.diii.2019.10.007

[20] van Erp, G. C. M. et al. Computational modeling of thermal ablation zones in the liver: A systematic review. *Cancers (Basel)*. **15**. 10.3390/cancers15235684 (2023). 10.3390/cancers15235684 PMC10705371 38067386

[21] Ruiter, S. J. S., Heerink, W. J. & de Jong K. P. Liver microwave ablation: a systematic review of various FDA-approved systems. *Eur. Radiol.* **29**, 4026–4035. 10.1007/s00330-018-5842-z (2019). 30506218 10.1007/s00330-018-5842-z PMC6611060

[22] Winokur, R. S. et al. Characterization of in vivo ablation zones following percutaneous microwave ablation of the liver with two commercially available devices: are manufacturer published reference values useful? *J Vasc Interv Radiol* **25**, 1939–1946 e. 10.1016/j.jvir.2014.08.014 (2014). 10.1016/j.jvir.2014.08.014 25307296

[23] Mulier, S. et al. Size and geometry of hepatic radiofrequency lesions. *Eur. J. Surg. Oncol.* **29**, 867–878 (2003). 14624780 10.1016/j.ejso.2003.09.012

[24] Pillai, K. et al. Heat sink effect on tumor ablation characteristics as observed in monopolar radiofrequency, bipolar radiofrequency, and microwave, using ex vivo calf liver model. *Med. (Baltim).* **94**, e580. 10.1097/MD.0000000000000580 (2015). 10.1097/MD.0000000000000580 PMC4553952 25738477

[25] Amabile, C. et al. Microwave ablation of primary and secondary liver tumours: ex vivo, in vivo, and clinical characterisation. *Int. J. Hyperth.* **33**, 34–42. 10.1080/02656736.2016.1196830 (2017). 10.1080/02656736.2016.1196830 27443519

[26] Siriwardana, P. N. et al. Effect of hepatic perfusion on microwave ablation zones in an ex vivo Porcine liver model. *J. Vasc Interv Radiol.* **28**, 732–739. 10.1016/j.jvir.2016.03.006 (2016). 27266361 10.1016/j.jvir.2016.03.006

[27] Chen, R., Zhang, J., Kong, D., Lou, Q. & Lu, F. Fast calculation of 3D radiofrequency ablation zone based on a closed-form solution of heat conduction equation fitted by ex vivo measurements. *Phys. Med. Biol.* **66**, 055022. 10.1088/1361-6560/abe052 (2021). 33503590 10.1088/1361-6560/abe052

[28] Ahmed, M. et al. Image-guided tumor ablation: standardization of terminology and reporting Criteria—A 10-Year update. *Radiology* **273**, 241–260 (2014). 24927329 10.1148/radiol.14132958 PMC4263618

[29] Kroeger, T. et al. Fast Estimation of the vascular cooling in RFA based on numerical simulation. *Open. Biomed. Eng. J.* **4**, 16–26. 10.2174/1874120701004020016 (2010). 20448794 10.2174/1874120701004020016 PMC2852120

[30] Berber, E. Laparoscopic microwave thermosphere ablation of malignant liver tumors: an initial clinical evaluation. *Surg. Endosc*. **30**, 692–698. 10.1007/s00464-015-4261-3 (2016). 26091998 10.1007/s00464-015-4261-3

[31] Berber, E. & Akbulut, S. Assessment of a new 150 W single-antenna microwave ablation system in the treatment of malignant liver tumors: the first worldwide experience. *J. Surg. Oncol.* **125**, 168–174. 10.1002/jso.26692 (2022). 34555190 10.1002/jso.26692

[32] Gemeinhardt, O. et al. Comparison of bipolar radiofrequency ablation zones in an in vivo Porcine model: correlation of histology and gross pathological findings. *Clin. Hemorheol. Microcirc.* **64**, 491–499 (2016). 27858704 10.3233/CH-168123

[33] Goldberg, S. N., Gazelle, G. S., Compton, C. C., Mueller, P. R. & Tanabe, K. K. Treatment of intrahepatic malignancy with radiofrequency ablation: radiologic-pathologic correlation. *Cancer* **88**, 2452–2463 (2000). 10861420

[34] Imajo, K. et al. New microwave ablation system for unresectable liver tumors that forms large, spherical ablation zones. *J. Gastroenterol. Hepatol.* **33**, 2007–2014. 10.1111/jgh.14294 (2018). 29851164 10.1111/jgh.14294

[35] Hui, T. C. H. et al. Microwave ablation of the liver in a live Porcine model: the impact of power, time and total energy on ablation zone size and shape. *Int. J. Hyperth.* **37**, 668–676. 10.1080/02656736.2020.1774083 (2020). 10.1080/02656736.2020.1774083 32552123

[36] Lehmann, K. S. et al. Minimal vascular flows cause strong heat sink effects in hepatic radiofrequency ablation ex vivo. *J. Hepatobiliary Pancreat. Sci.* **23**, 508–516. 10.1002/jhbp.370 (2016). 27338856 10.1002/jhbp.370

[37] Welp, C., Siebers, S., Ermert, H. & Werner, J. Investigation of the influence of blood flow rate on large vessel cooling in hepatic radiofrequency ablation. *Biomed. Tech. (Berl)*. **51**, 337–346. 10.1515/BMT.2006.067 (2006). 17155870 10.1515/BMT.2006.067

[38] Gaba, R. C. et al. Development and comprehensive characterization of Porcine hepatocellular carcinoma for translational liver cancer investigation. *Oncotarget* **11**, 2686–2701. 10.18632/oncotarget.27647 (2020). 32733642 10.18632/oncotarget.27647 PMC7367657

[39] Ortiz, C. B. et al. Creation of an Ex Vivo Renal Perfusion Model to Investigate Microwave Ablation. *J. Vasc. Interv. Radiol.* **34**, 40–45 e42. 10.1016/j.jvir.2022.10.013 (2023). 10.1016/j.jvir.2022.10.013 PMC12795510 36244634

[40] Ortiz, C. B. et al. Changes in Microwave Ablation Zone Dimensions after Transarterial Embolization in an Ex Vivo Human Kidney Perfusion Model. *J Vasc Interv Radiol* **35**, 1551–1557 e1551. 10.1016/j.jvir.2024.06.012 (2024). 10.1016/j.jvir.2024.06.012 PMC12908888 38901493

[41] Zurbuchen, U. et al. Determination of the electrical conductivity of human liver metastases: impact on therapy planning in the radiofrequency ablation of liver tumors. *Acta Radiol.* **58**, 164–169. 10.1177/0284185116639765 (2016). 27055920 10.1177/0284185116639765

[42] Tsuda, M. et al. Time-related changes of radiofrequency ablation lesion in the normal rabbit liver: findings of magnetic resonance imaging and histopathology. *Invest. Radiol.* **38**, 525–531. 10.1097/01.rli.0000073447.32361.b3 (2003). 12874519 10.1097/01.rli.0000073447.32361.b3

[43] Mulier, S. et al. Experimental and clinical radiofrequency ablation: proposal for standardized description of coagulation size and geometry. *Ann. Surg. Oncol.* **14**, 1381–1396 (2007). 17242989 10.1245/s10434-006-9033-9

[44] Goldberg, S. N., Gazelle, G. S. & Mueller, P. R. Thermal ablation therapy for focal malignancy: a unified approach to underlying principles, techniques, and diagnostic imaging guidance. *AJR Am. J. Roentgenol.* **174**, 323–331. 10.2214/ajr.174.2.1740323 (2000). 10658699 10.2214/ajr.174.2.1740323

[45] Brace, C. L., Diaz, T. A., Hinshaw, J. L. & Lee, F. T. Jr. Tissue contraction caused by radiofrequency and microwave ablation: a laboratory study in liver and lung. *J. Vasc Interv Radiol.* **21**, 1280–1286. 10.1016/j.jvir.2010.02.038 (2010). 20537559 10.1016/j.jvir.2010.02.038 PMC2920145

[46] Rossmann, C., Garrett-Mayer, E., Rattay, F. & Haemmerich, D. Dynamics of tissue shrinkage during ablative temperature exposures. *Physiol. Meas.* **35**, 55–67. 10.1088/0967-3334/35/1/55 (2014). 24345880 10.1088/0967-3334/35/1/55 PMC3924587

[47] Farina, L. et al. Characterisation of tissue shrinkage during microwave thermal ablation. *Int. J. Hyperth.* **30**, 419–428. 10.3109/02656736.2014.957250 (2014). 10.3109/02656736.2014.957250 25323026

[48] Poch, F. G. et al. The vascular cooling effect in hepatic multipolar radiofrequency ablation leads to incomplete ablation ex vivo. *Int. J. Hyperth.* **32**, 749–756. 10.1080/02656736.2016.1196395 (2016). 10.1080/02656736.2016.1196395 27400818

